# Cervical Chondrosarcoma Presenting as a Painless Neck Mass: A Case Report

**DOI:** 10.7759/cureus.107330

**Published:** 2026-04-19

**Authors:** Kimberly J Richardson, Russell Johnson, Lillian Chen, Sandhu Gurveen

**Affiliations:** 1 Internal Medicine, University of California Los Angeles David Geffen School of Medicine, Los Angeles, USA; 2 Internal Medicine and Pediatrics, University of California Los Angeles David Geffen School of Medicine, Los Angeles, USA

**Keywords:** cervical spine tumor, chondrosarcoma, en bloc resection, neck mass, primary bone malignancy, spinal oncology

## Abstract

Chondrosarcoma is a rare primary malignant tumor of the bone that typically presents with pain. Diagnosis may be challenging when patients present with a long-standing painless mass. We report a case of cervical chondrosarcoma to emphasize the importance of early recognition and management in optimizing outcomes. A 50-year-old man presented with a one-year history of a neck mass. Examination demonstrated a fixed, non-tender left posterior neck mass measuring approximately 5 x 2 cm without associated cervical, occipital, submandibular, submental, or supraclavicular lymphadenopathy. Imaging revealed an expansile lesion in the upper cervical spine without spinal canal involvement. Computed tomography (CT)-guided biopsy confirmed a low-grade cartilaginous neoplasm. The patient subsequently underwent a C2-3 laminectomy with en bloc tumor resection. Final pathology demonstrated low-grade chondrosarcoma with negative margins. There was no evidence of recurrence at 12-month follow-up. It is important to expand the differential for persistent neck masses, as early identification of chondrosarcoma facilitates definitive surgical management. Given the tumor's relative resistance to chemotherapy and radiation, complete surgical resection remains the foundation of treatment and the primary determinant of outcome.

## Introduction

Chondrosarcoma is a rare primary malignant bone tumor that mainly affects adults [1,2]. Its estimated incidence is one in 200,000 per year in the United States [1]. It is the second most common primary malignant bone tumor among primary bone neoplasms, with incidence increasing with age [2]. It most commonly affects adults, with a mean age at diagnosis of approximately 51 years, and the majority of cases occur in males after age 40 [1]. Patients may present with pain that is worse at night, a pathologic fracture, or an incidental finding [2]. Chondrosarcomas are characterized by indolent growth with symptoms present on average 10-15 months prior to diagnosis [1]. The majority of tumors are found in the extremities, followed by the axial skeleton [1]. Less than 15% of cases occur in the head and neck regions [3].

Subtypes of chondrosarcoma include conventional, dedifferentiated, mesenchymal, clear cell, and periosteal, with varying clinical behavior and prognosis [4]. The conventional subtype is the most common and is typically low grade, whereas higher-grade variants, such as dedifferentiated and mesenchymal chondrosarcoma, are associated with more aggressive disease [4]. Imaging, including CT and MRI, plays a pivotal role in diagnostic work-up; however, final pathology will determine the definitive diagnosis, as these tumors can be difficult to distinguish from benign cartilaginous lesions [5]. The gold standard of care remains surgical management withen bloc resection (complete removal of the tumor) and negative margins [1,2,5,6]. Here, we present a case of cervical spine chondrosarcoma to highlight the diagnostic challenge of distinguishing enchondroma from low-grade chondrosarcoma on limited biopsy, where clinical and radiographic correlation is essential for appropriate management.

## Case presentation

A 50-year-old man with a past medical history significant for human immunodeficiency virus (HIV), on bictegravir-emtricitabine-tenofovir, presented to the primary care clinic with a one-year history of a neck mass. The patient's HIV was well-controlled and not known to be associated with chondrosarcoma; therefore, it is considered an incidental finding in this case. He described the mass as hard and painless. He did not recall any specific injury. He denied numbness, tingling, weakness, fever, chills, night sweats, or unintentional weight loss. He endorsed limitations in neck mobility due to the size and location of the mass. Vital signs were as follows: blood pressure of 123/78 mmHg, heart rate of 83 beats per minute, temperature of 36.3 degrees Celsius, respiratory rate of 18 breaths per minute, oxygen saturation of 97% on room air, height of 5 ft 6 in (167.6 cm), and weight of 169 lbs (76.7 kg). The physical exam was remarkable for a fixed, non-tender left posterior neck mass measuring approximately 5 x 2 cm without associated cervical, occipital, submandibular, submental, or supraclavicular lymphadenopathy.

**Figure 1:**
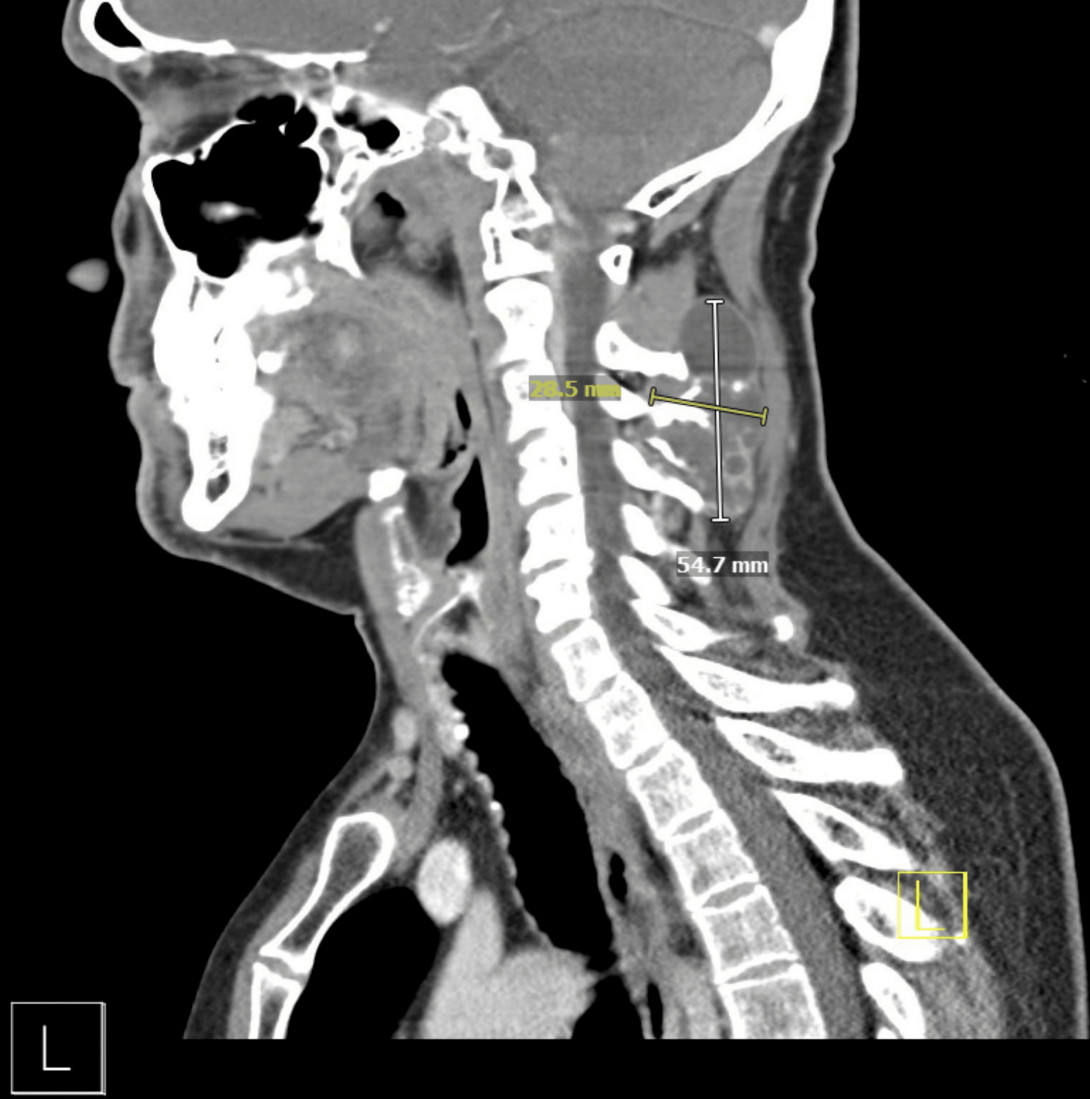
Contrast-enhanced CT of the neck. Sagittal image demonstrating an expansile multilocular cystic lesion centered within the C3 spinous process, measuring 28.5 mm x 54.7 mm.

Two days following presentation, a CT of the neck with contrast was obtained, which revealed an expansile multilocular cystic lesion centered within the C3 spinous process (Figure 1). 

**Figure 2:**
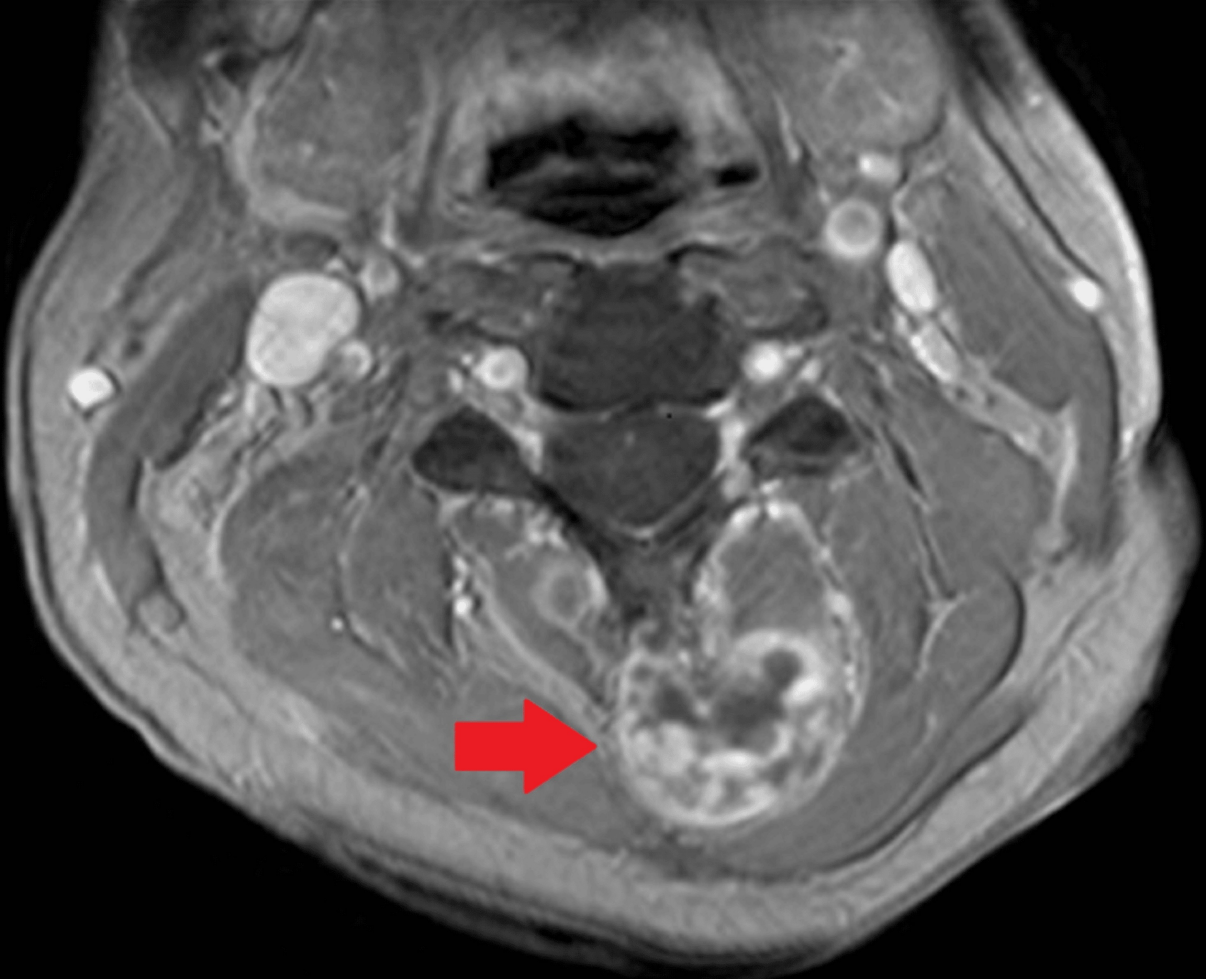
MRI of the cervical spine with contrast. Axial T1-weighted post-contrast image demonstrating heterogeneous enhancement of the tumor in the left lamina of C3 (red arrow).

Follow-up magnetic resonance imaging (MRI) of the cervical spine with and without contrast showed a multiloculated peripherally enhancing mass with internal nodular enhancing septations in the posterior elements of the upper cervical spine without spinal canal involvement (Figure 2).

He was referred to Neurosurgery Spine based on the lesion's location, associated structural involvement, and concern for malignancy, given the imaging findings.

**Figure 3:**
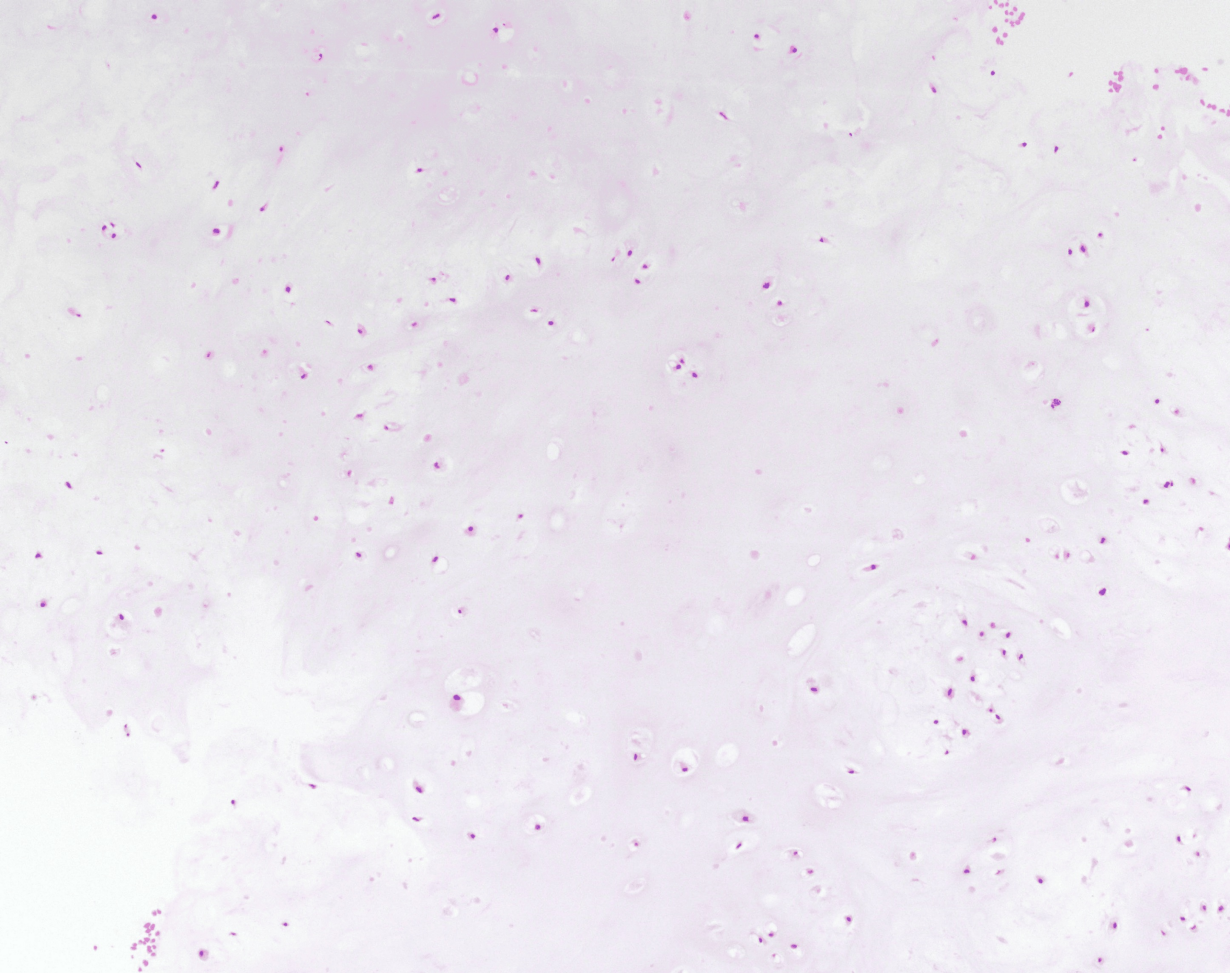
Histopathology from a CT-guided biopsy. Hematoxylin and eosin (H&E)-stained section (100x) demonstrating a proliferation of chondrocytic cells with uniform, small, round nuclei in a background of hyaline cartilage. No significant cytologic atypia, myxoid change, necrosis, or mitotic figures are identified. Findings are consistent with a low-grade cartilaginous lesion; however, distinction between enchondroma and low-grade chondrosarcoma is limited on a biopsy due to overlapping histologic features and sampling limitations.

Three weeks following presentation, he was evaluated by Neurosurgery Spine, at which time imaging was reviewed, and a CT-guided biopsy of the posterior cervical spine was recommended following a multidisciplinary tumor board discussion. A CT-guided cervical biopsy was performed without complications and with minimal estimated blood loss. Pathology showed a low-grade cartilaginous neoplasm, consistent with enchondroma (Figure 3).

However, given the location and patient's age, the possibility of a low-grade chondrosarcoma could not be entirely excluded.

**Figure 4:**
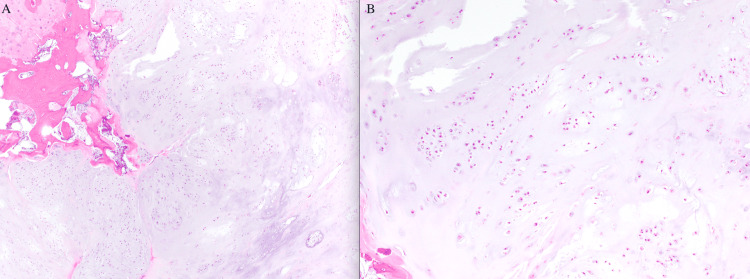
Histopathology from surgical resection. Hematoxylin and eosin (H&E)-stained sections demonstrating low-grade chondrosarcoma. (A) Low-power view (40x) showing lobulated hyaline cartilage with extension through the cortical bone and focal areas of nodular growth in the adjacent soft tissue. (B) High-power view (100x) demonstrating chondrocytes with uniform, small, round nuclei within lacunae and no significant cytologic atypia or mitotic figures.

Definitive surgical management with C2-3 laminectomy and en bloc tumor resection was performed four months after initial presentation, with an estimated blood loss of 80 cc and no intraoperative complications. The final pathology showed low-grade chondrosarcoma with negative margins (Figures 4A-4B).

He completed physical therapy and occupational therapy with improvement in neck mobility and upper body strength. At the 12-month follow-up, the patient remained without evidence of recurrence. His postoperative course was complicated by cervical radiculopathy and post-laminectomy kyphosis, ultimately requiring C3-6 anterior cervical discectomy and fusion.

## Discussion

Spinal chondrosarcomas account for approximately 2-12% of all chondrosarcomas, and a minority involve the cervical spine [6]. Patients commonly present with pain but may also endorse a palpable mass or neurologic symptoms [6]. Although pain is a commonly reported symptom, chondrosarcoma frequently presents as a slow-growing, painless mass, particularly in the head and neck [3]. Its indolent nature may contribute to delayed clinical evaluation, as seen in our patient with a one-year history of an asymptomatic neck mass.

Radiographs vary based on the histologic behavior of the tumor [6]. Imaging may reveal lysis and calcifications with the characteristic "rings and arcs" [6]. Plain radiographs are most often used for diagnosis, although CT and MRI are superior for evaluating disease extending into the surrounding soft tissue [4,6]. Open or CT-guided biopsies are required for histopathologic diagnosis [3]. Diagnosis and grading are challenging on biopsy, so treatment decisions are made by an interdisciplinary team [1,4].

Chondrosarcomas encompass several histologic subtypes, including conventional, dedifferentiated, mesenchymal, clear cell, and periosteal [4,5]. The conventional subtype accounts for more than 85% of cases [5]. Histologic diagnosis can be challenging when relying on core needle biopsy alone, as it may be difficult to differentiate between enchondroma and atypical cartilaginous tumors [4]. The presence of hyaline cartilage is a key histologic feature that distinguishes conventional chondrosarcomas from other primary bone malignancies [5]. In this case, histopathologic differentiation between enchondroma and low-grade chondrosarcoma was limited on a CT-guided biopsy, with definitive diagnosis established following surgical resection, demonstrating extension through the cortical bone and into the adjacent soft tissue. This emphasizes a well-recognized diagnostic challenge, as low-grade chondrosarcomas may closely resemble enchondromas on limited biopsy specimens. In such cases, clinical presentation and radiographic findings are critical in guiding management, particularly in axial lesions where a more aggressive approach is often warranted.

Surgical management withen bloc resection is the mainstay of treatment, as chondrosarcomas are largely resistant to chemotherapy and radiation [1,2,6]. This is mainly due to the tumor's slow growth and limited blood supply [1,4]. Achieving negative surgical margins is essential for minimizing locoregional recurrence and improving outcomes, with complete tumor excision representing an independent prognostic factor [1,4,6]. In this case, successful en bloc resection with negative margins was achieved, supporting this principle. Surgery may also play a role in metastatic conventional chondrosarcomas with potential benefits in overall survival and quality of life [4]. This is particularly relevant in patients with pulmonary metastases, as case series have shown improved prognosis with metastasectomy [4].

Although the lung is the most common site of metastasis, chondrosarcomas may also metastasize to other bones and soft tissue [1,4]. The more aggressive the subtype, the higher the risk of metastasis. Treatment options are limited for patients with advanced disease or metastatic spread, and there are no standard recommendations for chemotherapy [1]. Guidelines from the National Comprehensive Cancer Network (NCCN) and the European Society of Medical Oncology (ESMO) recommend adopting the established protocol for patients with Ewing's sarcoma [1,4]. The dedifferentiated subtype is particularly aggressive with a poor survival rate [1]. Given the limited efficacy and tolerability of chemotherapy, ongoing trials are investigating biologic therapies targeting the expressed protein PD-L1 [1]. These approaches are primarily relevant in advanced, unresectable, or dedifferentiated disease and are not applicable to low-grade lesions managed with complete surgical resection.

Close clinical follow-up and surveillance imaging are necessary for detecting local recurrence and metastasis early in the course of the disease [3]. Prognosis is very good for spinal chondrosarcomas, with a survival rate of 90% at five years for low-grade tumors [3]. In contrast, patients with metastatic disease have a survival rate of less than 30% at five years [1].

## Conclusions

Chondrosarcoma is the second most common primary malignant tumor of the bone. Our patient's presentation with a neck mass is atypical, as only a small portion of chondrosarcomas occur in the axial skeleton, and among those, the cervical spine is relatively infrequently involved. This case poses an important diagnostic challenge, as low-grade chondrosarcoma may resemble enchondroma on a limited biopsy. The absence of definitive malignant features on a biopsy should not exclude chondrosarcoma. Management decisions should incorporate clinical presentation and imaging findings and involve a multidisciplinary team. Complete surgical resection with negative margins remains the mainstay of treatment and is associated with favorable outcomes in low-grade disease.
